# Quick Diagnosis in Obstructive Sleep Apnea Syndrome: WatchPAT-200

**Published:** 2012-08-30

**Authors:** Tijen Ceylan, Hikmet Fırat, Gökhan Kuran, Sadık Ardıç, Esra Bilgin, Fatih Çelenk

**Affiliations:** 1 Department of Otorhinolaryngology, Yıldırım Beyazıt Education and Research Hospital, Ankara, Turkey; 2 Department of Pulmonary Medicine, Yıldırım Beyazıt Education and Research Hospital, Ankar, Turkey; 3 Department of Otorhinolaryngology, Gaziantep University Faculty of Medicine, Gaziantep, Turkey

**Keywords:** Sleep Apnea, Obstructive, Peripheral Arterial Disease

## Abstract

**Background:**

Obstructive sleep apnea syndrome (OSAS) is a highly prevelant disorder and found in approximately 2-4% of middle-aged adults.

**Objectives:**

To assess the efficacy of Watch-PAT-200 in the diagnosis of obstructive sleep apnea syndrome (OSAS).

**Patients and Methods:**

Patients suspected of having OSAS underwent overnight Level I polisomnography and simultaneously wore Watch-PAT-200 in the sleep laboratory.

**Results:**

51 adult patients included in the study. The average age was 45.3±10.5 years and the average body mass index (BMI) was 29.4±4.0 kg/m2. There was a high agreement between PSG and Watch PAT regarding apnea-hypopnea index, respiratory disturbance index and oxygen desaturation index. Significant but a low agreement was found in stage 1 and 2 of non-REM sleep when two methods compared. No agreement was found between PSG and Watch-PAT regarding stage 3 and 4 of non-REM sleep. Very low agreement was found between PSG and Watch-PAT regarding the REM sleep.

**Conclusions:**

Watch-PAT-200 is an effective method in the diagnosis of OSAS.

## 1. Background

Obstructive sleep apnea syndrome (OSAS) is a highly prevelant disorder and found in approximately 2-4% of middle-aged adults [1]. It is characterized by instability of the upper airway during sleep, which results in repetetive complete or partial collapse of the airway during sleep [1][2][3]. Apnea episodes are accompained by oxyhemoglobin desaturation [4]. OSAS is associated with various diseases including hypertension, coronary artery disease, congestive heart failure, myocardial infarction, cardiac sudden death, stroke, diabetes and metabolic abnormalities, motor vehicle accidents and mood disorders [5][6]. The excessive daytime somnolance interferes cognitive and social functioning [7][8][9]. Therefore, it is crucial to recognize clinically significant OSAS in order to prevent subsequent serious health problems.

Despite its high prevalence and significant consequences, many individuals with OSAS remain undiagnosed [10]. In-laboratory polisomnography (PSG) is the gold standard for diagnosing OSAS. PSG records several variables including airflow, abdominal movements, electroencephalography, and electromyography and oxygen saturation. However, PSG has many limitations including high cost, long waiting lists, limited availability and the need for technical expertise to perform and interpret [11]. Accordingly, it is not possible to perform PSG studies for all individuals suspected of having OSAS and in some countries waiting duration for PSG may exceed months to years [12]. Therefore, it is more plausible to find a simplier, cheaper and more accessable method for the diagnosis of OSAS. Home sleep studies have been considered an alternative method for the diagnosis of OSAS. Peripheral Arterial Tonometer (PAT) is a new method that is used in ambulatory diagnosis of OSAS. This technology uses a sensor that eliminates venous pulsations and continuously measures the arterial volume changes of the digit [13]. the arterial volume changes are regulated by α-adrenergic innervation and reflect the symphatetic activity [14]. Episodes of apnea and hypopnea cause arousals, sympathetic nervous system activation and peripheral vasoconstriction which may consequently result in attenuation of the PAT signal [15]. Watch-PAT-200 is a wrist-worn portable device that detects obstructive events by identifying the changes in sympathetic activity associated with the termination of the events. It has two finger probes and a main body. One of the probes contains a sensor that detects the PAT signal and the other probe measures arterial oxygen saturation. The main body of the device differentiates sleep time from wake time, carries out signal processing, provides the power supply and stores data. Watch-PAT-200 measures apnea-hypopnea index (AHI), respiratory disturbance index (RDI), total sleep time and sleep stages by using PAT signal.

## 2. Objectives

The aim of this study was to evaluate the efficacy of Watch-PAT-200 for diagnosing OSAS and determining the sleep stages by comparing its results to simultaneous PSG recordings.

## 3. Patients and Methods

Fifty-one adult patients with daytime sleepiness, habitual snoring and witnessed apnea referred to otolaryngology department of a tertiary referral center were included in the study. Patients with permanent cardiac pacemaker, cardiac arrythmia, peripheral vasculopathy or neuropathy, severe pulmonary disease, history of cervical or thoracic sympatectomy, finger deformities, history of the clinical use of α-adrenergic blockers and drug or alcohol addiction were excluded from the study. Following a thorough otolarygologic examination all patients underwent overnight level I PSG and simultaneously wore Watch-PAT in the sleep laboratory. The results were examined by two different sleep specialists independently.

### 3.1. Watch-PAT-200

For PAT signal recording Watch-PAT-200 (Itamar Medical Ltd., Caesarea, Israel) was used in this study. The physiologic signals recorded by the Watch-PAT included PAT signal, oxyhemoglobin saturation, sleep-wake states and heart rate which is derived from PAT signal. Apnea hypopnea index (AHI), respiratory disturbance index (RDI), oxygen desaturation index (ODI), total sleep time and sleep stage percentages were measured. All of the recorded signals are stored on a removable memory disk and downloaded to a computer for interpretation.

### 3.2. PSG

All patients underwent a standard in-laboratory overnight level I PSG. The recorded polysomnographic parameters included electroencephalogram, electrooculogram, chin and leg electromyogram, electrocardiogram. Thoracic and abdominal belts were used to determine respiratory efforts. Nasal cannula and thermistor for airflow, oxygen saturation, body position and snoring intensity were also recorded. Sleep was staged according to the standard criteria. An apnea was defined as complete cessation of for at least 10 seconds and hypopnea is scored if airflow was reduced by 50% or a lesser extent in association with a desaturation of at least 3% or an arousal. Apnea and hyponea index (AHI) was calculated by dividing the total number of apnea and hypopnea events by the total sleep time. A respiratory disturbance index (RDI) was calculated as the total number of respiratory events divided by the sleep time. An oxygen desaturation index was calculated as the number of oxygen desaturations of at least 4% per hour of sleep.

### 3.3. Ethical Considerations

Informed consent was obtained from all participants to wear the Watch-PAT device simultaneously with the PSG. The Institutional review board approved the study.

### 3.4. Statistical Analysis

Data analysis was performed by using SPSS for Windows, version 11.5 (SPSS Inc., Chicago, IL, United States). Data were shown as mean ± standard deviation. The agreement between the PSG and Watch-PAT-200 was assessed by calculated intra-class correlation and Bland-Altman plots. Receiver operating characteristic (ROC) analysis was carried out to evaluate the Watch-PAT-200 diagnostic capability. A threshold of PSG-AHI > 15 was used as the cut-off point for OSAS diagnosis. Based on these threshold definitions, ROC curves were derived and areas under the curves (AUCs) were calculated. A p value less than 0.05 was considered statistically significant.

## 4. Results

The study group consisted of 51 adult patients with suspected of having OSAS. The avarage age was 45.3 ± 10.5 years, ranging between 27 and 71 years. The average body mass index (BMI) was 29.4 ± 4.0 kg/m2. The mean PSG AHI was 27 ± 23.4 (1.5-78.4), while the mean Watch-PAT AHI was 29.6 ± 20.9 (2.3-71.1). There was a high agreement between PSG AHI and Watch-PAT AHI (ICC = 0.941; 95% confidence interval (CI) = 0.899-0.966, P < 0.001). The mean PSG RDI was 28.1±23.2 (1.5-80.0), while mean WacthPAT RDI was 32.1 ± 19.5 (3.8-71.3). A high agreement was found between PSG RDI and Watch-PAT RDI (ICC = 0.916; 95% CI = 0.858-0.951, P < 0.001). The mean ODI derived from PSG was 19.6±20.0 (0.6-83.4), while the mean ODI derived from Watch-PAT was 20.4 ± 19.5 (0-63.84). There was a high agreement between PSG ODI and Watch-PAT ODI (ICC = 0.877; 95% CI = 0.794-0.928, P <0.001). According to the data derived from both methods, significant but low agreement was found in stage 1 and 2 of non-REM sleep between PSG and Watch-PAT (ICC = 0.495, 95% CI = 0.258-0.676, P < 0.001). There was no agreement in stage 3 and 4 of non-REM sleep between two methods (P = 0.514). There was a very low agreement between PSG and Watch-PAT regarding the REM sleep (ICC = 0.237, 95% CI = 0.-0.478, P = 0.044). The comparison of the parameters obtained by both PSG and Watch-PAT-200 was given in (Table 1). If mild OSAS patients are compared with moderate and severe ones according to AHI, sensivity, spesivitiy, positive predictive value, negative predictive value and diagnostic accuracy are % 93.1, % 66.1, % 79.4, % 84.6 and % 80.8. If mild and moderete OSAS patients are compared with severe ones according to AHI, sensivity, spesivitiy, positive predictive value, negative predictive value and diagnostic accuracy are % 88.2, % 80.0, % 71.4, % 92.3 and % 83.0. (Table 2)

**Table 1:** Comparison of the Parameters Obtained by Both PSG anD Watch-PAT-200

	**AHI**	**RDI**	**ODI**
PSG	27 ± 23.4	28.1 ± 23.2	19.6 ± 20.0
Watch-PAT	29.6 ± 20.9	32.1 ± 19.5	20.4 ± 19.5

**Table 2:** Comparison of the Parameters Obtained by Both PSG anD Watch-PAT-200

	**Sensitivity**	**Specificity**	**Positive Predictive Value**	**Negative Predictive Value**	**Diagnostic Accuracy**
AHI < 15	93.1 %	66.1 %	79.4 %	84.6 %	80.8 %
AHI < 30	88.2 %	80.0 %	71.4 %	92.3 %	83.0 %

## 5. Discussion

Watch-PAT-200 analyzes the attenuation of PAT signal, increased heart rate and decreased oxygen saturation [16]. PAT signal, actigraphy and blood oxygen levels are used to measure AHI, RDI, total sleep time and sleep stage percentages. In addition, it is also possible to determine whether the low oxygen saturation is due to an obstructive or a central event [17].

There are studies supporting the validity of Watch-PAT in the diagnosis of OSAS. Schnall et al. [13] found a highly significant correlation between standard AHI scoring and PAT-vasoconstriction events. Ayas et al. [14] evaluated the correlation between the PSG and the Watch-PAT in 30 patients and found that there was a significant correlation and good agreement of AHI between two methods. Bar et al. [16] found a high correlation and good agreement between PSG AHI and PAT AHI. Pittman et al. [18] found a high correlation of AHI and ODI between PSG and Watch-PAT. Pang et al. [17] reported a good correlation of AHI and lowest oxygen saturation (LSAT) between the PSG and the Watch-PAT. O’Donnell et al. [15] induced experimental upper airway obstruction and concluded that airflow obstruction in patients with OSAS results in PAT signal attenuation.

In this study we assessed the diagnostic value of Watch-PAT-200 to detect OSAS by comparing standart in-laboratory PSG results. Watch-PAT and the PSG were performed simultaneously on the same night in order to eliminate the night-to-night variation. The parameters including AHI, ODI, RDI and REM and non-REM sleep stages derived from PSG and Watch-PAT recordings were compared. We found a high agreement of important respiratory parameters during sleep such as AHI, ODI and RDI between PSG and Watch-PAT. When mild OSAS patients are compared with moderate and severe ones according to AHI, sensivity, specificity, positive predictive value, negative predictive value and diagnostic accuracy were 93.1 %, 66.1 %, 79.4 %, 84.6 % and 80.8 %, respectively. When mild and moderate OSAS patients are compared with severe ones according to AHI, sensivity, specificity, positive predictive value, negative predictive value and diagnostic accuracy were 88.2 %, 80.0 %, 71.4 %, 92.3 % and 83.0 %, respectively.

Sleep stages are mainly divided into two phases: REM sleep and non-REM sleep. REM sleep is most often associated with vivid dreaming and a high level of brain activity and non-REM sleep is usually associated with reduced neuronal activity [19]. Sympathetic and parasympathetic activities show certain changes with sleep stages. Sympathetic activation is often increased in REM sleep relative to non-REM sleep. Thus, high sympathetic activity may help to identify REM sleep. Increased sympathetic activation correponds to PAT signal attenuation and helps to identify REM sleep [20]. In this study we attempted to score REM and non-REM sleep by using Watch-PAT-200. Significant but a low agreement was found between PSG and Watch-PAT regarding the REM sleep. There was no agreement between PSG and Watch-PAT regarding stage 3 and 4 of non-REM sleep. Finally, significant but a low agreement was found in stage 1 and 2 of non-REM sleep between two techniques. Watch-PAT 200 may give an idea about sleep architecture of the OSAS patients.

There are several limitations of this study. Because the Watch-PAT cannot measure airflow, it cannot differentiate hypopneas from apneas. The application of the device was performed in laboratory rather than at home. Testing at home may give different results. Finally, although the use of the device is simple, some patients may have difficulty in placing the the device accurately on their wrist and fingers.

Watch-PAT-200 is a small, cheap and safe device which is used in the diagnosis of OSAS. In this study Watch-PAT demonstrated similar results with the PSG in respiratory sleep parameters including AHI, RDI and ODI. However, we found significant but low and no agreement in sleep stages between two methods. As a conclusion, Watch-PAT-200 is an effective and a reliable method in the diagnosis of OSAS.
